# Potential Mechanisms of the Improvement of Glucose Homeostasis in Type 2 Diabetes by Pomegranate Juice

**DOI:** 10.3390/antiox11030553

**Published:** 2022-03-15

**Authors:** Carlos Olvera-Sandoval, Héctor Enrique Fabela-Illescas, Eduardo Fernández-Martínez, María Araceli Ortiz-Rodríguez, Raquel Cariño-Cortés, José Alberto Ariza-Ortega, Juan Carlos Hernández-González, Diana Olivo, Carmen Valadez-Vega, Helen Belefant-Miller, Gabriel Betanzos-Cabrera

**Affiliations:** 1 Facultad de Medicina Mexicali, Universidad Autónoma de Baja California, Mexicali 21000, Mexico; olvera.carlos@uabc.edu.mx; 2 Programa de Enfermedades Cardiometabólicas, Jurisdicción Sanitaria II Tulancingo, Servicios de Salud de Hidalgo, Pachuca 43679, Mexico; fa146593@uaeh.edu.mx; 3 Laboratory of Medicinal Chemistry and Pharmacology, Centro de Investigación en Biología de la Reproducción, Área Académica de Medicina, Instituto de Ciencias de la Salud, Universidad Autónoma del Estado de Hidalgo, Pachuca 42090, Mexico; efernan@uaeh.edu.mx (E.F.-M.); raquel_carino4897@uaeh.edu.mx (R.C.-C.); 4 Facultad de Nutrición, Universidad Autónoma del Estado de Morelos, Mexico Iztaccíhuatl 100 Col. Los Volcanes, Morelos, Cuernavaca 62350, Mexico; araceli.ortiz@uaem.mx; 5 Área Académica de Nutrición, Instituto de Ciencias de la Salud, Universidad Autónoma del Estado de Hidalgo, Ex-Hacienda de la Concepción, Tilcuautla, Pachuca de Soto 42080, Mexico; jose_ariza@uaeh.edu.mx (J.A.A.-O.); diana_olivo@uaeh.edu.mx (D.O.); 6 Área Académica de Medicina Veterinaria y Zootecnia, Instituto de Ciencias Agropecuarias, Universidad Autónoma del Estado de Hidalgo, Av. Universidad km 1 Exhacienda de Aquetzalpa A.P. 32, Tulancingo 43600, Mexico; juan_hernandez8281@uaeh.edu.mx; 7 Área Académica de Medicina, Instituto de Ciencias de la Salud, Universidad Autónoma del Estado de Hidalgo, Ex-Hacienda de la Concepción, Tilcuautla, Pachuca de Soto 42080, Mexico; maria_valadez2584@uaeh.edu.mx; 8 Dale Bumpers National Rice Research Center, Stuttgart, AR 72160, USA; drhelenmiller@gmail.com; 9 Área Académica de Nutrición y Medicina, Instituto de Ciencias de la Salud, Universidad Autónoma del Estado de Hidalgo, Ex-Hacienda de la Concepción, Tilcuautla, Pachuca de Soto 42080, Mexico

**Keywords:** type 2 diabetes, hypoglycemic, polyphenols, pomegranate juice

## Abstract

Pomegranate is a polyphenol-rich fruit. Studies have shown that extracts prepared from its juice or from different parts of the pomegranate plant have various biological activities including antioxidant, antimicrobial, anti-inflammatory, anticarcinogenic, cardioprotective, and antidiabetic. The therapeutic potential has been attributed to various phytochemicals, including ellagic acid, punicic acid, flavonoids, anthocyanidins, anthocyanins, flavonols, and flavones. This review focuses on the scientific evidence of pomegranate juice as hypoglycemic, emphasizing the chemical composition and the possible mechanisms of action associated with this effect. Studies were identified using the PubMed, Scopus, and ISI Web of Science databases to identify relevant articles focused on the hypoglycemic effect of pomegranate juice. The physiological responses to pomegranate juice are reported here, including a decrease of oxidative stress damage, an increase of insulin-dependent glucose uptake, maintenance of β-cell integrity, inhibition of nonenzymatic protein glycation, an increase of insulin sensitivity, modulation of peroxisome proliferator-activated receptor-gamma, inhibition of α-amylase, inhibition of α-glucosidase and dipeptidyl peptidase-4, and decreases in inflammation. Overall, we found a significant hypoglycemic effect of pomegranate in in vitro and in vivo studies and we summarize the potential mechanisms of action.

## 1. Introduction

Pomegranate (*Punica granatum* L.) belongs to the Lythraceae family, a small family of trees and shrubs, 1.5 to 5 m high, with irregular spiny branches and bright leaves [1]. Trees up to 200 years old have been reported [2]. The fruit is a berry, measuring 5–12 cm in diameter and with a rounded hexagonal shape covered by a thick layer (peel) that protects the edible part and weighing approximately 200 g [3]. The fruit is native to Central Asia, from Iran and Turkmenistan to northern India, the Mediterranean, and the Middle East [2]. During the conquest, Spanish sailors took the fruit to Mexico, north California in the United States, and other places of Latin America [4].

Different cultures have specifically given the fruit importance and value for centuries. The Egyptians included it as part of the fruit supply for the Pharaoh’s residence (about 1600 BC) [5]. The Babylonians considered the fruit a resurrection agent, the Persians believed it gave them invincibility on the battlefields, while in ancient China, the seeds symbolized longevity and immortality [6]. In Judaism, it is said that the number of seeds of a single fruit is 613, which represents the 613 commandments of the Torah. Buddhism considers the pomegranate as one of the three blessed fruits and it is associated with fertility. Quran, the sacred scripture of Islam, describes a heavenly paradise where pomegranates are to be found [4]. Currently, in Spain, the pomegranate is used as an emblem on the national flag.

**Figure 1:**
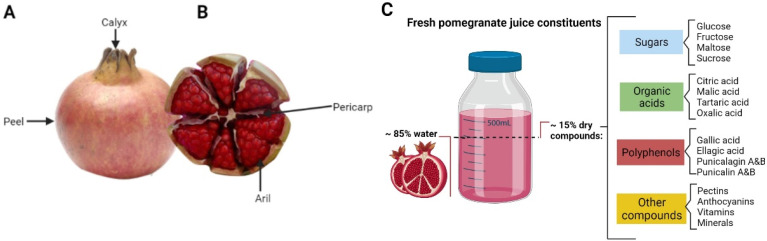
Pomegranate fruit and its constituents. (**A**) External identification of pomegranate parts like calyx and exocarp named peel. (**B**) Internal parts of fruit organized by arils enveloped a pericarp layer. (**C**) Fresh pomegranate juice constituents. Created in Biorender.com.

In the pomegranate fruit, the peel is about 30% of the fresh weight of the fruit Figure 1A, and the ratio of weights of the peel, arils, and seeds is 50:40:10, respectively [7]. As shown in Figure 1B, the edible part consists of prismatic-shaped arils with internal seeds and is surrounded by the pericarp, a white membrane that separates and protects them [8]. The aril’s juice is intense red due to the anthocyanins contained in the juice. Variations in color intensity among different cultivars is mainly due to the concentration of these compounds [9].

## 2. Pomegranate Juice Composition

The 15% dry matter from pomegranate juice corresponds to sugars, organic acids, pectins, vitamins, and minerals. The juice is particularly rich in antioxidant polyphenols, including hydrolyzable tannins, condensed tannins, anthocyanins, flavonols, and phenolic acids, as shown in Figure 1C [10]. The polyphenol compounds have been studied for health benefits against different diseases [9,11]. Punicalagins are hydrolyzable tannins and are the most abundant polyphenol in juice, responsible for over 50% of the potential antioxidant of juice [12]. After digestion, punicalagins are hydrolyzed into ellagic acid, and after further metabolism by the intestinal microbiota, converted into urolithins and their conjugates found in blood and urine [13].

Carbohydrate metabolism breaks down complex sugars into glucose, which serves as the primary metabolic fuel for the body. Poor glucose metabolism leads to diabetes, a metabolic disorder of carbohydrate metabolism characterized by high blood glucose levels resulting from insufficient insulin production or an ineffective response of cells to insulin.

The glycemic index is a number assigned to foods based on how quickly and how high those foods cause increases in blood glucose levels and as a measure of how much glucose is released into the blood from food. A low glycemic index indicates a slow and steady release of glucose, whereas a high glycemic index indicates a rapid release of glucose. The slow and steady release of glucose helps maintain reasonable glucose control and pomegranate juice is helpful in promoting a low glycemic index. Kerimi et al. [14] showed that pomegranate polyphenols in pomegranate juice could reduce bread’s acute postprandial glycemic response (bread is absorbed quickly and causes a quick rise in blood glucose). It was proposed that pomegranate punicalagin inhibits α-amylase, causing sugars to be released more gradually, thus preventing spikes in sugar levels.

## 3. Pharmacological Treatments against Type 2 Diabetes

In the last 30 years, diabetes has established itself as a global public health problem, affecting urbanized regions significantly more than rural areas. It represents one of the 10 leading causes of death from its complications. Currently, for every confirmed case of diabetes, others are not detected, and the number of people with this disease is expected to rise sharply in the following years. [15].

In the absence of insulin secretion, as occurs in people with type 1 diabetes, the standard treatment is the administration of exogenous insulin. The primary treatments for type 2 diabetes are hypoglycemic drugs. Pharmacological treatments for the control and management of type 2 diabetes have emerged to reduce hyperglycemia and its complications. Table 1 shows a list of oral hypoglycemics acting as gluconeogenic inhibitors, pancreatic insulin secretion stimulants, glucose absorption inhibitors, glucose reuptake blockers, and/or insulin sensitizers [16].

The most appropriate treatment for the management and control of diabetes lies in multidisciplinary intervention: promoting lifestyle changes through healthy eating and regular exercise, accompanied by a correct pharmacological prescription that minimizes the dose and the adverse effects that a prolonged pharmacological treatment can cause. In this context, there is a deep and constant interest in studying phytochemical compounds present in foods that can provide therapeutic or preventive effects to deal with diseases whose usual management has been pharmacological. These bioactive compounds are safe, have few adverse effects, and have been proposed as complementary treatments to delay or decrease the impact of several diseases such as diabetes, cancer, and hypertension [23].

## 4. Fruit Derived Bioactive Compounds with Hypoglycemic Activity

Fruits and other natural sources have bioactive compounds with pharmacological activity and, so far, fewer undesirable effects. With the emergence of increasingly well-structured studies showing the therapeutic properties of bioactive compounds in fruits, there has been renewed interest in using these natural sources not as a definitive treatment but as a complementary treatment [24].

The fruits of particular scientific interest are those with compounds that have color and that usually have high antioxidant activity. However, in recent years it has been demonstrated that their function is not limited to reducing oxidative stress, but that they also exert effects at the cellular level such as control of the opening and closing of channels, inhibition-activation of enzymes, secretion of hormones, and interactions with DNA, the cell cycle, and inflammatory processes [25]. Some of the bioactive compounds in fruits with antidiabetic activity are polyphenols, terpenoids, saponins, carotenoids, alkaloids, and glycosides. It has also been established that these compounds can have a therapeutic effect in their isolated form and even a synergistic effect between them, enhancing an expected response [26]. The fruits and vegetables’ antidiabetic activity has been tested with extracts obtained by maceration and liquefaction using chemical solvents, water extraction, products obtained by food preservation techniques, or simply fresh juice.

**Figure 2:**
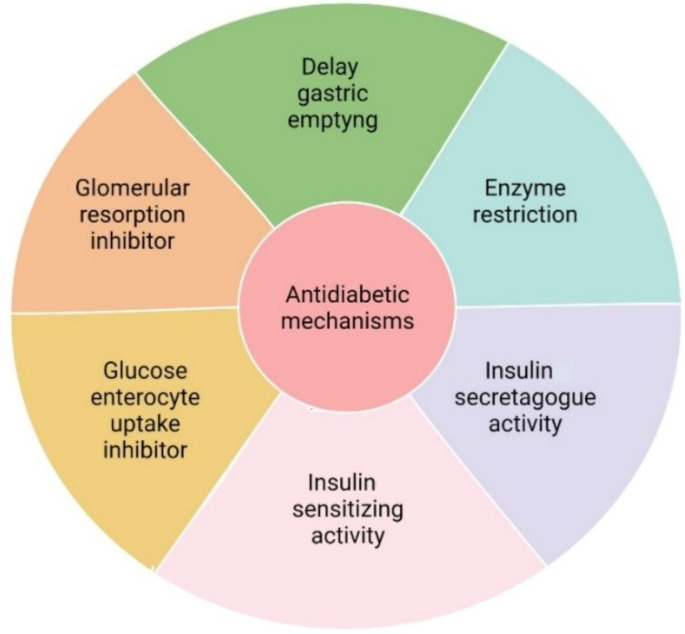
Primary mechanisms of action that are considered for the study of hypoglycemic activity.

It is important to identify the diverse mechanisms of action for treating type 2, which are briefly summarized in Figure 2 [27]. The mechanisms of action are summarized:

(1) Delayed gastric emptying through inhibition of dipeptidyl peptidase 4 (DPP-4) and glucagon-like peptide 1 (GLP-1);
(2) Inhibition of α-amylase and α-glucosidase, both carbohydrate digesting enzymes, and inhibition of hepatic glucose release;
(3) Insulin secretagogue activity, stimulating pancreatic β-cells function;
(4) Enhancing glucose uptake by stimulating of expression of GLUT transporters in muscle, liver, and adipose cells;
(5) Decrease glucose absorption by enterocytes by inhibiting sodium-glucose transport (SGLT-1) and GLUT-2 transporters;
(6) Reduction of glomerular glucose reuptake followed by low glucose levels in the blood.

## 5. Antidiabetic Effects of Fresh Pomegranate Juice

Experimental designs using cell cultures, animal models, and pre-clinical trials have been used to evaluate the health effects of fresh pomegranate juice [28]. The results of studies on the hypoglycemic effects of pomegranate juice in animals and humans are not uniform, as some show hypoglycemic activity in animals [29,30] and humans [31,32,33], while others show no hypoglycemic activity effect [34,35,36] (Table 2).

**Figure 3:**
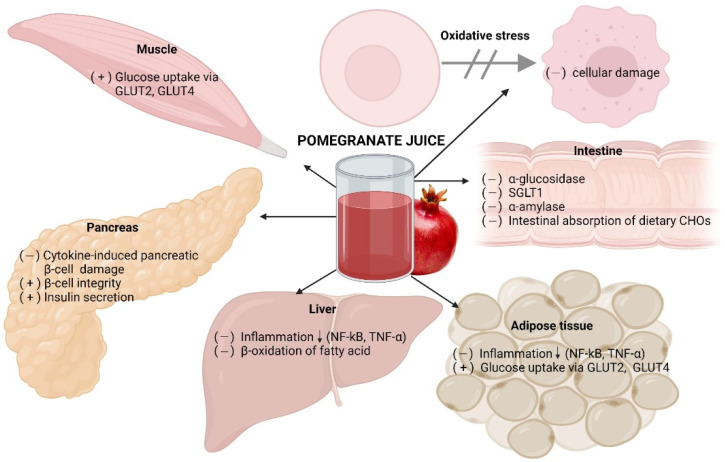
Potential mechanisms of pomegranate juice or its extracts to improve glucose homeostasis.

Diverse studies have evaluated pomegranate juice and its components on type 2 diabetes and established possible mechanisms of action. A high glycemic condition leads to protein glycation, beginning with the nonenzymatic binding of reducing sugar or sugar derivatives to the protein’s amine group, leading to advanced glycation end products. These products play a role in the pathology of chronic human diseases, including type-2 diabetes, through two primary mechanisms: (1) Activating cell signaling leading to the production of reactive oxygen species (ROS) and inflammatory factors [45,46] and (2) degrading the extracellular matrix, leading to a loss in its function of structural support for the cells and tissues, as shown in Figure 3 [47,48]. This condition facilitates the production of radical oxygen species, establishing oxidative stress in all organs, including the pancreas, which promotes the development of type 2 diabetes [49,50]. Hence, the high antioxidant capacity of pomegranate juice’s unique components, such as the predominant punicalagin, may delay type 2 diabetes by neutralizing the generated reactive oxygen species directly. The juice may also act indirectly through the induction of the expression of antioxidant enzymes. Pomegranate juice increased the activity of paraoxonase 1 (PON1), an enzyme with antioxidant activity [31] and increased pon 1 gene expression in streptozotocin-induced diabetic mice fed with a high-fat diet [51].

### 5.1. Human Studies

Pomegranate juice significantly reduced serum lipid peroxides and thiobarbituric acid reactive substances levels by 56% and 28%, respectively, in 10 healthy subjects and 10 non-insulin-dependent diabetes mellitus patients. Additionally, PON1 activity increased dramatically, by 24% [52]. Pomegranate juice consumption did not affect serum glucose, cholesterol, or triglyceride levels. The results suggest that PJ consumption had anti-oxidative effects in diabetic patients, though no decrease in diabetic parameters was apparent [52].

Esmaillzadeh et al. [53] demonstrated that after 8 weeks of pomegranate juice concentrate consumption by 22 diabetic patients, plasma lipid profiles were improved, as evidenced by decreased total cholesterol LDL-cholesterol levels, and a decreased LDL/HDL ratio. However, there were no significant changes in serum triacylglycerol and HDL-cholesterol concentrations [53]. Shishehbor et al. reported that 40 type 2 diabetes patients consuming pomegranate juice concentrate for 4 weeks produced a significant increase in both total and high-density lipoprotein cholesterol and a significant reduction in serum interleukin-6. However, changes in serum triglyceride, low-density lipoprotein cholesterol, fasting blood glucose, and blood pressure were not statistically significant [38].

In healthy subjects, the consumption of reconstituted pomegranate juice decreased the postprandial glycemic response after consumption of high glycemic index food more than a polyphenol-rich extract of pomegranate juice did. The work suggested that pomegranate juice polyphenols could reduce the postprandial glycemic response of bread. Additionally, punicalagin was an effective in vitro inhibitor of human α-amylase to the drug acarbose, a drug which acts by inhibiting human α-amylase [54,55], while ellagic acid and punicalin were less effective [14].

In healthy and diabetic subjects who received a single oral dose of 1.5 mL of fresh pomegranate juice/kg body weight during fasting, the peripheral insulin increased significantly at 1 and 3 h after the consumption. This indicates that the consumption of fresh pomegranate juice can be used as a complementary treatment for patients with hyperglycemia and as a prophylactic agent [37].

### 5.2. Rodent Studies

Tugcu et al. [42] demonstrated the antioxidant properties of pomegranate juice by using a rat model of diabetes. Diabetes-induced rats were daily administrated with pomegranate juice for 10 weeks, and the treatment significantly reduced the levels of 8-hydroxy-2′-deoxyguanosine and malondialdehyde (a marker of oxidative DNA damage and an indicator of lipid peroxidation, respectively). Likewise, glutathione levels were reduced and glutathione peroxidase (a ROS scavenger) activity was increased following the administration of pomegranate juice. Interestingly, no statistically significant difference was found for levels of another ROS scavenger, superoxide dismutase [42]. Mohan et al. [43] also showed that pomegranate juice supplementation (100 mg/kg for four weeks) reduced oxidative stress in diabetic Wistar rats. Kidney and pancreas tissues had lower thiobarbituric acid reactive substances and higher antioxidant enzymes (superoxide dismutase, catalase, and glutathione reductase) [43].

Streptozotocin-nicotinamide-induced diabetes in male Sprague-Dawley rats was treated orally with pomegranate juice for 21 days. Diabetic rats showed reduced activities of antioxidant enzymes such as total antioxidant status, superoxide dismutase, and catalase. In contrast, the levels of biomarkers of oxidative stress such as gamma-glutamyl transferase and malondialdehyde increased in diabetic control rats compared to normal control rats. These results demonstrated that pomegranate protects against oxidative stress [56].

Pomegranate juice improved the structure of the pancreas in male Wistar rats with streptozotocin-induced hyperglycemia; ad libitum consumption of pomegranate juice for 26 days resulted in hypoglycemic effects and improved conservation of pancreatic architecture post-mortem [57]. Taheri Rouhi et al. [58] reported the protective role of pomegranate juice on the architecture and preservation of Langerhans islets. In streptozotocin-nicotinamide-induced type 2 diabetic Sprague–Dawley rats, the pomegranate juice administration significantly reduced plasma total cholesterol, triglycerides, and LDL levels. Although a non-significant reduction of plasma glucose levels was found, pomegranate juice significantly improved Langerhans’ size and the number of islets compared with diabetic rats. These results suggest that pomegranate juice compounds with high antioxidant properties are responsible for their anti-hyperlipidemic and anti-inflammatory effects and restoration effect on the damaged islets of Langerhans [58].

Fresh pomegranate juice also acts synergistically with oral hypoglycemics to provide an antioxidant environment that minimizes complications of diabetes. Alloxan-induced diabetic rats treated with tolbutamide (a sulfonylurea) mixed with pomegranate juice proved to have better hypoglycemic control than a group that received only tolbutamide, as evidenced by higher serum insulin levels and lower glycemia. The research concluded, through pharmacokinetic studies, that the bioactive compounds in the pomegranate could inhibit cytochrome P450 2C9, which is responsible for the breakdown of sulfonylureas (the oldest class of oral antidiabetic medication) [30].

Sugar-containing polyphenolic anthocyanins from pomegranate decreased oxidative stress in macrophages. Rozenberg et al. [59] demonstrated that pomegranate juice sugar consumption by diabetic mice for 10 days resulted in a slight but significant decrement in their peritoneal macrophage total peroxide levels and an increment in cellular glutathione content. These antioxidant effects could be due to the presence of unique complex sugars or phenolic sugars in pomegranate juice that can scavenge free radicals and inhibit a Fenton reagent •OH generating system, possibly by chelation of a ferrous ion [59].

Resistin is a molecule secreted by adipocytes during the differentiation of adipocytes. It is known that fatty mass in obesity is strongly associated with type 2 diabetes [60]. Thus, resistin neutralization improves adipocyte insulin-stimulated glucose uptake, ameliorating obesity-induced insulin resistance [61]. The pomegranate aril is rich in punicic acid, a conjugated alpha-linolenic acid with immunomodulatory and anti-inflammatory properties [62]. Through PPAR-γ modulation, punicic acid can inhibit various pro-inflammatory cytokines such TNF-α, IL-6, IL-8, and IL-12 [63]. Moreover, ellagic acid, the main component of pomegranate juice and other colored fruits such as strawberry, black raspberry, and blackberry [64], reduced the serum resistin levels in ovariectomized mice (animal model with elevated resistin levels). Similarly, a pomegranate extract and ellagic acid decreased the intracellular resistin levels in differentiated murine 3T3-L1 adipocytes [65].

### 5.3. In Vitro Studies

Pomegranate polyphenols like ellagic acid and punicalagin appear to have inhibitory effects on the activity of α-amylase, α-glucosidase, and dipeptidyl peptidase-4 in free-cell systems and adipocyte cell lines. The polyphenols act as α-amylase inhibitors like the drug acarbose [54,55]. Bellesia et al. [64] demonstrated that ellagic acid and punicalagin (exclusive to pomegranate) inhibited α-glucosidase [64]. Les et al. [54] showed that pomegranate juice ellagic acid and punicalagin inhibited α-glucosidase and DPP-4, and lipase. These two polyphenols, as well as urolithin A (a metabolite generated during the metabolism of pomegranate ellagitannins by intestinal microbiota), also significantly reduced triglyceride accumulation and gene expression related to adipocyte formation [54]. Overall, the study showed that punicalagin, ellagic acid, and urolithin A could inhibit enzymes in carbohydrate-triglyceride-related metabolisms, such as GLUT4, DPP-4, and lipase.

Transcription factors are associated with type 2 diabetes progression. The nuclear factor-kappa B (NF-κB) controls various genes involved in inflammation and is associated with diabetic nephropathy [66]. Increased cellular levels of reactive oxygen species activated PPAR-γ, which led to the up-regulation of genes implicated in mediating expression of fat-specific genes and in activating the program of adipocyte differentiation, causing an increase in disease progression [56]. In vitro studies with vascular-endothelial cells showed that pomegranate wine was a potent inhibitor of NF-κB activation [67,68]. Pomegranate juice may inhibit NF-κB activation, diminishing the development of type 2 diabetes complications, especially cardiovascular complications.

Another transcriptional factor affected by pomegranate is the peroxisome proliferator-activated receptor gamma (PPAR-γ), which regulates fatty acid storage, adipocyte differentiation, and glucose metabolism. PPAR-γ is a target of antidiabetic drugs. PPAR-γ activators are widely used in the treatment of type 2 diabetes (Table I) because they improve the sensitivity of insulin receptors. Quercetin, a flavonoid found in pomegranate, showed PPAR γ binding activity. These results suggest that PPAR- γ is a molecular target for pomegranate compounds and represents a potential mechanism for the anti-diabetic action of fruit. [69,70]. Gallic acid (another phenol prominent in juice) can also have this binding activity [71]. Similarly, pomegranate seed oil increased the PPARγ expression, inhibiting colonic tumors in chemically induced colon carcinogenesis rats. [72]. Diverse pomegranate compounds including catechin, ellagic acid, myricetin, kaempferol, quercetin, and gallic acid have PPAR binding activity [69,70].

Shiner et al. [73] demonstrated that pomegranate juice’s PPAR γ -mediated in vitro effect reduces oxidative stress in macrophages; this effect was caused by PPAR γ g inhibition; the same effect was observed after macrophages were incubated with punicalagin and gallic acid from pomegranate [73].

## 6. Conclusions

Pomegranate juice has a substantial variety of bioactive compounds with potential action against metabolic disorders, including type 2 diabetes. As suggested by in vitro and in vivo studies, several mechanisms for glucose homeostasis through multiple modes of action have been proposed for the activity, including increased antioxidant activity, an increase in the function of B-cells, glucose uptake via GLUT2 and GLUT4, lower activity of a-amylase enzyme, reduction of glucose absorption by inhibiting the enzyme α-glucosidase and dipeptidyl peptidase-4, absolutely smashing the sensitivity of insulin receptors with increased activity of the peroxisome proliferator-activated gamma receptor (PPAR-γ). Thus, pomegranate and its compounds may represent an adjuvant for the current treatments against type 2 diabetes, supporting their therapeutic effects and reducing their side effects by reducing their dosage. Nevertheless, it is still necessary to address more clinical trials to measure their effectiveness by considering variables such as comorbidities, pharmacological interaction, and dietary patterns.

## Figures and Tables

**Table 1:** Classification of the main oral hypoglycemic medications used in the treatment of diabetes.

Oral Hypoglycemic Medications	Mechanism of Action	Main Adverse Effects	Reference
Sulfonylureas	Binds to K-ATP channels leading to the influx of Ca^++^ and thus increasing insulin secretion by pancreatic beta cells.	Dizziness, nervousness, diarrhea, dyspepsia, and headache.	[17]
Meglitinides	Binds at different pancreatic beta-cell receptors increasing influx of Ca^++^ promoting the releasing of insulin.	Hypoglycemia, headache, and respiratory infections.	
Biguanides	Reduces hepatic gluconeogenesis, decreases intestinal absorption of glucose, and increases insulin sensitivity of muscle cells.	Diarrhea, nausea and vomiting, flatulence, and headache.	[18]
Thiazolidinediones	Promotes genic expression at different levels by binding to PPAR-γ receptors which improves insulin sensitivity, decreases hepatic glucose production, and mediates the inflammatory response.	Edema, hypoglycemia, cardiac failure, and headache.	[19]
α-Glucosidase inhibitors	Decreases glucose absorption at the intestinal level by competitively inhibiting α -glucosidase.	Flatulence, diarrhea, and abdominal pain.	[20]
SGLT2 inhibitors	Prevents glomerular glucose reabsorption by inhibiting the sodium-glucose cotransporter 2 (SGLT-2).	Urinary tract infections, glycosuria, dyslipidemia, and hyperphosphatemia.	[21]
DPP-4 inhibitors	Inhibits protease activity of DPP-4 by prolonging the release of glucagon; furthermore, it reduces gastric emptying and improves insulin secretion.	Hypoglycemia, headache, and urinary tract infections.	[22]

**Table 2:** Effect of pomegranate juice and its extracts on type 2 diabetes using in vivo and vitro models.

Study	Derivative	Model	Dose	Effect	Reference
Pomegranate juice, but not an extract, confers a lower glycemic response on a high glycemic index	Pomegranate juice	Healthy human	200 mL in 4 days	Reduced blood glucose	[14]
Effect of pomegranate juice on paraoxonase enzyme activity in patients with type 2 diabetes	Pomegranate juice	Humans with type 2 diabetes mellitus	200 mL for 6 weeks	Reduced concentration of fasting blood sugar and paraoxonase and increased arylesterase activity of PON1	[31]
Fresh pomegranate juice decreases fasting serum erythropoietin in patient with type 2 diabetes	Pomegranate juice	Humans with type 2 diabetes mellitus	3 h afer administration of 1.5 mL per kg afer a 12-h fast	Reduced serum erythropoietin level	[37]
Effects of concentrated pomegranate juice on subclinical inflammation and cardiometabolic risk facto for type 2 diabetes:	Concentrated pomegranate juice	Humans with type 2 diabetes mellitus	50 g for 4 weeks	Caused significant reduction in serum interleukin-6 (IL-6). However, fasting blood glucose changes were not statistically significant.	[38]
Study of the antidiabetic activity of *Punica granatum* L. fruits aqueous extract on the alloxan-diabetic wistar rats	Aqueous extract of pomegranate arils	Alloxan induced diabetic rats	100, 200, 350 mg/kg for 21 days	Reduced modulation of hyperglycemia	[39]
Mechanism of pomegranate ellagic polyphenols reducing insulin resistance on gestational diabetes mellitus rats	Pomegranate ellagic polyphenols	STZ induced diabetic rats with gestational diabetes mellitus	50, 150, 300 mg/(kg/day) for 14 days.	Reduced of blood glucose levels, blood biochemical index, and insulin resistance.	[40]
Effects of pomegranate aril juice and its punicalagin on some key regulators of insulin resistance and oxidative liver injury in streptozotocin-nicotinamide type 2 diabetic rats	Pomegranate juice and its punicalagin	STZ induced diabetic rats	100 or 300 mg/kg and 2.6 or 7.8 mg/kg respectively for 6 weeks.	Reduced insulin resistance and hyperglycemia. Low dose of punicalagin induced some modulation, nevertheless, the high dose of punicalagin did not show any antidiabetic activity	[41]
Protective effect of pomegranate juice on retinal oxidative stress in streptozotocin-induced diabetic rats	Pomegranate juice	STZ induced diabetic rats	100 µL for 10 weeks	Reduced the levels of 8-hydroxy-2′-deoxyguano-sine (8OHdG) and malondialdehyde	[42]
Effect of pomegranate juice on angiotensin II-induced hypertension in diabetic wistar rats	Pomegranate seed extract	STZ induced diabetic rats	100, 300 mg/kg for 4 weeks	Reversed the biochemical changes induced by diabetes	[43]
Effect of punicalagin on multiple targets in streptozotocin/high-fat diet induced diabetic mice	Punicalagin	STZ induced diabetic mice	100, 150, 200 mg/kg for 4 weeks	Reduced of blood glucose levels and had a protective effect on diabetes mellitus	[44]

## Abbreviations

CYP2C9	Cytochrome P450 2C9
DPP-4	Dipeptidyl peptidase 4
GLP-1	Glucagon-like peptide 1
GLUT2	Glucose transporter 2
GLUT4	Glucose transporter 4
HDL	High-density lipoprotein
Il	Interleukin
LDL	Low-density lipoprotein
NF-κB	Nuclear factor-kappa B
•OH	Hydroxyl radical
PON1	Paraoxonase 1
PPAR-γ	Peroxisome proliferator-activated receptor gamma
SGLT-1	Sodium glucose transporter 1
TNF-α	Tumor Necrosis Factor alpha
